# The number of osteoclasts in a biopsy specimen can predict the efficacy of neoadjuvant chemotherapy for primary osteosarcoma

**DOI:** 10.1038/s41598-020-80504-w

**Published:** 2021-01-21

**Authors:** Yoshihiro Araki, Norio Yamamoto, Katsuhiro Hayashi, Akihiko Takeuchi, Shinji Miwa, Kentaro Igarashi, Takashi Higuchi, Kensaku Abe, Yuta Taniguchi, Hirotaka Yonezawa, Sei Morinaga, Yohei Asano, Hiroko Ikeda, Takayuki Nojima, Hiroyuki Tsuchiya

**Affiliations:** 1 Department of Orthopaedic Surgery, Graduate School of Medical Sciences, Kanazawa University, 13-1, Takaramachi, Kanazawa-city, Ishikawa 920-8641 Japan; 2 Department of Pathology, Kanazawa University, Kanazawa, Japan

**Keywords:** Oncology, Surgical oncology, Oncogenesis, Mechanisms of disease, Bone cancer, Cancer microenvironment, Sarcoma

## Abstract

Osteosarcoma is the most common primary malignant bone tumor, and its standard treatment is a combination of surgery and chemotherapy. A poor response to chemotherapy causes unfavorable oncological outcomes. We investigated the correlation between osteoclast differentiation in biopsy specimens and the efficacy of neoadjuvant chemotherapy in resected specimens. Forty-nine patients who underwent neoadjuvant chemotherapy and subsequent surgical treatment at our institution between 1999 and 2018 were enrolled. Using medical records, we investigated the age, sex, tumor size, location, subtype, staging, chemotherapy agents (doxorubicin, cisplatin, ifosfamide, and methotrexate), number of neoadjuvant chemotherapy courses, number of osteoclasts in biopsy specimens, and efficacy of neoadjuvant chemotherapy according to the Rosen and Huvos classification (Grade I-IV) in resected specimens. Univariate and multivariate analyses were performed to identify factors predictive of a good response in resected specimens after neoadjuvant chemotherapy. A good response (Grade III/IV) was detected in 25, while a poor response (Grade I/II) was detected in 24. According to the multivariate analysis, ≥ 46 years old (odds ratio [OR], 0.05; 95% confidence interval [CI], 0.01–0.45; p < 0.01) and ≥ 5 mature osteoclasts in a biopsy specimen (OR, 36.9; 95% CI, 6.03–225; p < 0.01) were significantly associated with the neoadjuvant chemotherapy efficacy. The accuracy for predicting a good response to chemotherapy based on ≥ 5 osteoclasts in a biopsy specimen in patients < 46 years old was 85%. The number of mature osteoclasts in biopsy specimens is a simple factor for predicting the efficacy of chemotherapy before treatment, although further studies will be required to determine the underlying mechanism.

## Introduction

Osteosarcoma is the most common primary malignant bone tumor, occurring predominantly in children, adolescents, and young adults, but sometimes in elderly people as well^1^. The standard treatment for osteosarcoma is a combination of surgery and chemotherapy^2^. A poor response to chemotherapy in osteosarcoma patients regularly causes an unfavorable oncological outcome^3^.

Osteosarcoma in elderly people shows a second incidence peak, but the prognoses are reported to be poor. One reason for such poor prognoses is due to a low sensitivity to chemotherapy, with an efficacy rate ranging from 0 to 48%^4,5^, and thus, surgical treatment alone is usually performed without neoadjuvant chemotherapy, due to the susceptibility of adverse events occurring in association with chemotherapy.

Osteosarcoma has several subtypes according to the 2020 WHO classification^1^, among which the chondroblastic-type is reported to demonstrate a poor response to chemotherapy, with a rate of 20–43% of the usual efficacy due to the difficulty of delivering agents inside the chondroblastic components^6–8^. However, neoadjuvant chemotherapy for the chondroblastic-type is usually performed to reduce micrometastasis, in addition to shrinking of the tumor to improve the accessibility of surgical treatment, as well as for other subtypes of osteosarcoma.

In general, a good response to chemotherapy is observed in tumor cells with brisk mitosis or hypervascularity before chemotherapy^3^. However, the histological findings predictive of the efficacy of neoadjuvant chemotherapy in osteosarcoma cells are cytologic anaplasia and positive p16 expression, which differ from the high rate of mitosis and tumor necrosis in osteosarcoma^9–11^. Furthermore, a few novel markers, such as M2TA and miR221, have been reported to be early predictive biomarkers for the efficacy of neoadjuvant chemotherapy in osteosarcoma patients in recent years^12–14^. However, little is known about the correlation between the histological findings of osteoclasts around osteosarcoma cells and the efficacy of neoadjuvant chemotherapy.

Osteoclasts are created by the fusion of three or more macrophages. The role of macrophages in tumor treatment remains unclear; however, a type of tumor-associated macrophage was reported to enhance or limit the efficacy of chemotherapy^15–18^. Osteoclast differentiation was found to be dependent on the condition of macrophages^19^.

Osteoclast differentiation reflects the good condition of tumor-associated macrophages, and the presence of mature osteoclasts may be predictive of a good response to chemotherapy. We therefore investigated the correlation between the osteoclast differentiation in biopsy specimens and the efficacy of neoadjuvant chemotherapy in resected specimens.

## Results

### Patient characteristics

The study population included 31 men and 18 women. Their median age at the diagnosis was 23 (range 5–70) years old. The location of the tumor was the extremities in 45 cases and trunk in 4 cases. The average tumor size was 91 mm in the greatest dimension (range 53–237 mm). The subtypes of primary osteosarcoma were osteoblastic type in 23 cases, chondroblastic type in 6 cases, fibroblastic type in 2 cases, telangiectatic type in 2 cases, small-cell type and giant-cell-rich type in 1 case each, and unknown type in 14 cases. The staging according to the AJCC 8th edition was Stage IIA in 21 cases, StageIIB in 21 cases, and StageIVB in 7 cases. The number of courses of DOX (60 mg/m^2^/course) as neoadjuvant chemotherapy was 7 in 2 cases, 6 in 7 cases, 5 in 19 cases, 4 in 4 cases, 3 in 8 cases, 2 in 6 cases, and 1 in 2 cases. The number of courses of CDDP (120 mg/m^2^/course) as neoadjuvant chemotherapy was 8 in 1 case, 7 in 1 case, 6 in 7 cases, 5 in 18 cases, 4 in 6 cases, 3 in 8 cases, 2 in 6 cases, and 1 in 1 case. The number of courses of IFO (9 g/m^2^/course) as neoadjuvant chemotherapy was 6 in 2 cases, 5 in 2 cases, 4 in 2 cases, 3 in 6 cases, 2 in 5 cases, and 1 in 5 cases. The number of courses of MTX (12 g/m^2^/course [< 10 years of age], 10 g/m^2^/course [10–20 years old], 8 g/m^2^/course [> 20 years of age]) as neoadjuvant chemotherapy was 5 in 1 cases, 3 in 2 cases, and 1 in 2 cases. The average total number of Cathepsin K-positive osteoclasts with ≥ 3 nuclei in 10 different fields at 40 times magnification power in each biopsy specimen was 10 (range 0–50).

### ROC curve analyses for the optimal cut-off values

The optimal cut-off values were as follows: age, 46 years old(area under the curve [AUC], 0.59); size, 93 mm (AUC, 0.53); number of osteoclasts in the biopsy specimen, 5 (AUC, 0.75), number of courses of DOX as neoadjuvant chemotherapy, 3 (AUC, 0.63); number of courses of CDDP as neoadjuvant chemotherapy, 3 (AUC, 0.65); number of courses of IFO as neoadjuvant chemotherapy, 4 (AUC, 0.51), and number of courses of MTX as neoadjuvant chemotherapy, 1 (AUC, 0.48) (Table 1).

**Table 1:** The optimal cut-off values of factors by ROC curve.

Factors	Cut-off value	AUC	95%CI
Age (years old)	46	0.588	0.423–0752
Size (mm)	93	0.525	0.354–0.696
Osteoclast number	5	0.745	0.597–0.893
**Course number**			
DOX	3	0.633	0.481–0.785
CDDP	3	0.65	0.499–0.801
IFO	4	0.511	0.36–0.661
MTX	1	0.476	0.389–0.563

DOX: doxorubicin, CDDP: cisplatin, IFO: ifosfamide, MTX: methotrexate, ROC: receiver operating characteristics, AUC: area under curve, CI: confidence intervals.

The efficacy of chemotherapy according to the Rosen and Huvos classification, was as follows: Grade I, n = 6; Grade II, n = 18; Grade III, n = 14; and Grade IV, n = 11. The number of patients with a good response (Grade III/IV) was 25, and the number of the patients with a poor response (Grade I/II) was 24.

### Univariate and multivariate analyses for identifying factors

In the univariate analysis, age ≥ 46 years old, size ≥ 93 mm, ≥ 5 osteoclasts in the biopsy specimen, ≥ 3 courses of chemotherapy using DOX, and ≥ 3 courses of chemotherapy using CDDP were significantly associated with the efficacy of neoadjuvant chemotherapy (Table 2). In the multivariate analysis, age (odds ratio [OR], 0.05; 95% confidence interval [95% CI], 0.006–0.45; p < 0.01) and the number of osteoclasts (OR, 36.9; 95% CI, 6.03–225; p < 0.01) were significantly associated with the efficacy of neoadjuvant chemotherapy (Table 3). Only 2 patients over 46 years old showed a good response to chemotherapy. Twenty-one of the 27 patients with ≥ 5 Cathepsin K-positive osteoclasts showed a good response to chemotherapy, while 18 of the 22 patients with < 5 Cathepsin K-positive osteoclasts showed a poor response to chemotherapy (Table 4).

**Table 2:** The univariate analysis of factors for predicting the good response of chemotherapy.

Factors	n	OR	95%CI	*p* value
**Age**				
≥ 46 years old	9	0.211	0.039–1.15	0.07
< 46 years old	40			
**Sex**				
Male	31	1.07	0.33–3.41	0.91
Female	18			
**Size**				
≥ 93 mm	15	3.73	0.98–14.2	0.05
< 93 mm	33			
**Location**				
Trunk	4	3.14	0.30–32.5	0.34
Extremity	45			
**Stage**				
III/IV	7	2.62	0.46–15.1	0.28
I/II	41			
**Subtype**				
Chondroblastic type	6	0.16	0.02–1.47	0.11
Other types	43			
**Osteoclast number**				
≥ 5	27	15.7	3.83–64.7	< 0.01
< 5	22			
**Course number**				
DOX				
≥ 3	40	4.74	0.87–25.7	0.07
< 3	9			
CDDP				
≥ 3	41	9.88	1.11–87.9	0.04
< 3	8			
IFO				
≥ 4	7	1.33	0.27–6.7	0.73
< 4	42			
MTX				
≥ 1	5	0.61	0.09–4.01	0.61
< 1	44			

DOX: doxorubicin, CDDP: cisplatin, IFO: ifosfamide, MTX: methotrexate, OR:odds ratio, CI: confidence intervals.

**Table 3:** The multivariate analysis for predicting the good response of chemotherapy.

Factors	n	OR	95%CI	p-value
**Age**				
≥ 46 years old	9	0.05	0.006–0.45	< 0.01
< 46 years old	40			
**Osteoclast number**				
≥ 5	27	36.9	6.03–225	< 0.01
< 5	22			

OR :Odds ratio, CI : Confidence intervals.

**Table 4:** Cross-classification of OS patients by IHC of CathepsinK and efficacy of neoadjuvant chemotherapy.

	Efficacy of neoadjuvant chemotherapy		Total
	Grade I/II (poor response)	Grade III/IV (good response)	
**IHC of cathepsin K**			
Osteoclast number ≥ 5	6	21	27
Osteoclast number < 5	18	4	22
Total	24	25	49

OS : osteosarcoma, IHC : immunohistochemical staining.

### Patients demographic data according to the number of osteoclast

The 27 patients had ≥ 5 Cathepsin K-positive osteoclasts and the 22 patients had < 5 Cathepsin K-positive osteoclasts in a biopsy specimen. The factor of age, tumor size, and the course number of each agent used for neoadjuvant chemotherapy did not significantly differ in the two groups by analyzing using a non-paired student’s t test. The factor of sex, tumor location, stage, and subtype were not also significantly different in the two groups by analyzing using a chi-squared test (Table 5).

**Table 5:** Patients demographic data according to the number of osteoclast.

	IHC of cathepsin K		
	Osteoclast number ≥ 5	Osteoclast number < 5	
N	27	22	-
**Age**			
Mean ± SD	24.1 ± 20.6	22.0 ± 14.5	0.68
**Sex**			
Male	16	15	0.52
Female	11	7	
**Size [mm]**			
Mean ± SD	88.7 ± 40.7	87.2 ± 21.2	0.63
**Location**			
Trunk	2	2	0.83
Extremity	25	20	
**Stage**			
III/IV	3	4	0.48
I/II	24	18	
**Subtype**			
Chondroblastic type	2	4	0.25
Other types	25	18	
**Course number (mean)**			
DOX	4.6	3.7	0.05
CDDP	4.7	3.7	0.05
IFO	1	1.7	0.17
MTX	0.15	0.41	0.37

DOX: doxorubicin, CDDP: cisplatin, IFO: ifosfamide, MTX: methotrexate, SD: standard deviation.

### The accuracy analysis

The accuracy in predicting a good response to chemotherapy based on the number of osteoclasts in biopsy specimen in patients < 46 years was 85% (sensitivity, 83%; specificity, 88%; positive likelihood ratio, 7.02; and negative likelihood ratio, 0.197) (Table 6).

**Table 6:** Predictive accuracy measures.

	Estimate	95% CI	
		Lower limit	Upper limit
Positive ratio	0.525	0.361	0.685
True positive ratio	0.575	0.409	0.73
Sensitivity	0.826	0.612	0.95
Specificity	0.882	0.636	0.985
Positive predictive value	0.905	0.696	0.988
Negative predictive value	0.789	0.544	0.939
Predictive accuracy	0.85	0.702	0.943
Positive likelihood ratio	7.022	1.885	26.16
Negative likelihood ratio	0.197	0.08	0.488

CI : confidence intervals.

## Discussion

Numerous studies on osteosarcoma have revealed that a good response to neoadjuvant chemotherapy was significantly associated with a good survival^2–14^. However, osteosarcoma in elderly people and chondroblastic-type osteosarcoma are commonly chemo-resistant, with 0%-48% of cases showing a good response to neoadjuvant chemotherapy^4–8^. The pretreatment identification of cases in whom chemotherapy will be effective is essential to obtain a good outcome; however, there are few methods for identifying patients who may show a good response to neoadjuvant chemotherapy before treatment^9–14^. In recent years, several novel biomarkers, including MT2A, GLS1, and miRNA-221, have been reported to be useful for the early prediction of the response to neoadjuvant chemotherapy^12–14^. However, these biomarkers are difficult to measure in daily examinations. If a good response to chemotherapy can be easily predicted at the diagnosis of osteosarcoma, chemotherapy should be urgently performed for the patients.

In elderly patients, the efficacy of chemotherapy is not usually good due to their decreased renal function, cardiac dysfunction, and decreased bone marrow tolerance. In previous studies, good responders were observed in 0%-48% of cases > 40 years old who received neoadjuvant chemotherapy^3^. In our study, only 2 of 9 patients (22%) over 46 years old showed a good response to neoadjuvant chemotherapy, and age < 46 years old was a predictor of a good response to chemotherapy as demonstrated in previous studies^4,5^ (Table 3). The chemotherapy regimen, dose, and the number of courses differed among elderly patients, mainly due to the frequency of adverse events and organ dysfunction with aging, and the efficacy of chemotherapy depended on individual patients’ characteristics^4,5^. However, the multivariate analysis in our study revealed that, rather than the type of drug or number of courses, the age and osteoclast number were predictors of the efficacy of neoadjuvant chemotherapy. Both of the elderly patients with a good response of chemotherapy showed ≥ 5 Cathepsin K-positive mature osteoclasts in biopsy specimens, and thus, even in elderly patients, a good efficacy of neoadjuvant chemotherapy might be expected when the biopsy specimens showed ≥ 5 Cathepsin K-positive mature osteoclasts, although the number of patients investigated was very small.

Osteosarcoma has several subtypes according to the 2020 WHO classification^1^, and among which the chondroblastic-type is reported to be relatively resistant to chemotherapy, with a rate of 20%-43% of the usual efficacy due to the difficulty of delivering agents inside the chondroblastic components^6–8^. In this study, there were six patients with chondroblastic-type osteosarcoma, however, two patient showed a good response of neoadjuvant chemotherapy, and their biopsy specimen histology revealed the presence of ≥ 5 Cathepsin K-positive mature osteoclasts. The number of investigated patients was very small, however, according to these findings, neoadjuvant chemotherapy might be effective even in patients with chondroblastic type osteosarcoma, when biopsy specimens demonstrated the presence of ≥ 5 Cathepsin K-positive mature osteoclasts.

The role of macrophages in tumor treatment remains unclear; however, a type of tumor-associated macrophage was reported to enhance or limit the efficacy of chemotherapy^15–18^. A previous study reported that, in soft tissue sarcoma patients whose biopsy specimens contained large number of CD68-positive macrophages, the resected specimen had few viable tumor cells, suggesting a good response to neoadjuvant chemotherapy^18^.

Drug-carrier particles are regularly taken in by the macrophage phagocyte system and concentrated in bone tissues. A good drug delivery to bone tissue can be determined based on the presence of good phagocytosis of drug-carrier particles by bone marrow macrophages^20^. In the bone microenvironment, osteoclasts are produced from the fusion of 3–100 macrophages, and osteoclast differentiation is regularly found to be dependent on the condition of macrophages, which are osteoclast precursors^15,16^. In our study, ≥ 5 Cathepsin K-positive mature osteoclasts in a biopsy specimen was associated with a good response to neoadjuvant chemotherapy, suggesting that osteoclast differentiation around osteosarcoma cells reflects a good condition of tumor-associated macrophages and a well-working drug delivery system into bone marrow. Thus, the presence of the mature osteoclasts in a biopsy specimen may help predict a good response to neoadjuvant chemotherapy.

Little is known about the role of osteoclasts around osteosarcoma cells, including determining the efficacy of chemotherapy, although the osteoclasts exist in the microenvironment of bone tumors^15,16^. A previous report stated that when the number of osteoclasts around osteosarcoma cells in a biopsy specimen is decreased, the prognosis is worsened because of the increased rate of lung metastasis^21,22^. However, the cause and mechanism underlying this phenomenon remain unclear. Our study suggests that chemosensitivity to osteosarcoma cells may be better when osteoclast differentiation is retained in the bone microenvironment around the osteosarcoma cells. High-grade malignancy with a chemo-resistant nature might contribute to the suppression of osteoclastogenesis, although further basic research is necessary to validate these findings.

The effects of zoledronate, an inhibitor of bone absorption by osteoclasts, on osteosarcoma cells have been confirmed both in vitro and in vivo in numerous studies; these effects include the inhibition of cell growth, induction of apoptosis, and reduction of metastasis^23^. However, according to the outcomes of phase III clinical trials in France and China, zoledronate combined with standard chemotherapy for osteosarcoma patients is not recommended, as the overall survival (OS) and event-free survival (EFS) were shown to be decreased in both trials^24,25^. Osteoclasts, in addition to macrophages, surrounding osteosarcoma cells disappear when zoledronate is used with chemotherapy; as a result, the drug delivery system to the bone tissues cannot function well, and the effects of chemotherapy may be reduced. In the present study, similar outcomes regarding the OS and EFS according to the presence or absence of osteoclasts were observed (*P*-values; 0.07 and 0.05) (Supplementary Fig. S1A, B). However, the confirmation of similar outcomes was not a novel approach to investigate the cause of a poor OS and EFS in previous clinical trials. We therefore evaluated the efficacy of chemotherapy as an outcome measure for the existence of osteoclast differentiation or the presence of mature osteoclasts in a biopsy specimen, rather than the survival of OS and EFS.

The present study was associated with some limitations. First, this was a retrospective study of a relatively small study population in a single institution. Second, the same regimen of chemotherapy before surgery was not administered to all patients. Third, the biopsy specimen was a part of the bone lesion, and thus may not reflect all aspects of the histological nature. However, the bone microenvironment around the osteoid produced by the tumor cells was observed in this study, and the actual changes in the bone microenvironment, including osteoclast differentiation, could thus be clearly seen.

In conclusion, counting the number of mature osteoclasts in a biopsy specimen is a simple method of predicting the efficacy of chemotherapy before treatment, although further studies will be required to validate the mechanism underlying the phenomenon.

## Methods

### Patients

One hundred and thirty-one patients with osteosarcoma were treated at our institution between 1999 and 2018. All data were acquired though the pathological database. Among them, cases in which a biopsy was performed at another institution (n = 46), cases with another subtype of osteosarcoma(e.g. secondary or low-grade osteosarcoma) (n = 11), and cases in which the amount of material in the biopsy specimen was not sufficient to prepare an additional tissue section for immunohistochemical (IHC) staining (n = 4) were excluded from this study. The remaining 70 cases underwent an open biopsy at our institution, and a diagnosis of primary osteosarcoma was made.

Among these 70 cases, a total of 49 patients who underwent neoadjuvant chemotherapy and subsequent surgical treatment at our institution were enrolled in this study.

This retrospective study of patient specimens was approved by the ethical committee of Kanazawa University Hospital (Institutional Review Board Number 2019–217(3249)) in compliance with the guidelines of the 1975 Declaration of Helsinki, and written informed consent was obtained from all study participants and/or their parents (in case of children).

### Patient characteristics data collection

The following variables were investigated based on medical records: age, sex, tumor size (greatest dimension), tumor location, subtype, staging, chemotherapy agents (doxorubicin [DOX], cisplatin [CDDP], ifosfamide [IFO], and methotrexate [MTX]), number of neoadjuvant chemotherapy courses, number of osteoclasts in the biopsy specimen, and the efficacy of neoadjuvant chemotherapy in the resected specimen. The tumor size was defined as the length in the greatest dimension measured on computed tomography (CT) or magnetic resonance imaging (MRI). The tumor location was classified as trunk or appendicular site. The subtypes of osteosarcoma were determined according to 2020 WHO classification^1^. The staging was defined according to TNM classification system in the AJCC 8th edition^26^. The number of osteoclasts around the tumor cells in open biopsy specimens before treatment was counted, using Cathepsin K staining, and positivity was defined by a receiver operating characteristics(ROC) curve analysis for the total number of osteoclasts with ≥ 3 nuclei in 10 different fields using microscope at 40 times magnification power. The Cathepsin K-positive cells were counted using BZ X Analyzer software program (KEYENCE, Osaka, Japan). The efficacy of chemotherapy was determined according to the Rosen and Huvos classification. The grade was assigned Grade I-IV according to the percentage of tumor necrosis in the resected specimen.

### Immunohistochemical staining

For the fixation of biopsy specimen, 10% formaldehyde (Muto Pure Chemicals Co., Ltd., Tokyo, Japan) was used at room temperature. After being immersed in formaldehyde for 24 h, paraffin-embedded specimens were made by Tissue-Tek VIP 6AI (Sakura Finetek Japan Co.,Ltd., Tokyo, Japan). Four-micrometer-thick sections cut from the representative block of each tumor were deparaffinized. The preparations were autoclaved in citrate buffer (pH 6.0), and endogenous peroxidase activity was blocked with 3% hydrogen peroxide. The primary antibody of Cathepsin K (sc-48353 [E-7], dilution 1:200; SANTA CRUZ BIOTECHNOLOGY, INC, Dallas, Texas, USA) was used. Slides were incubated for 1 h at room temperature with the primary antibody, and subsequently labelled using the secondary antibody (K4063, Envision + Dual Link System-HRP, Dako, Carpinteria, California, USA). The sections were examined using BZ X710 microscope (KEYENCE, Osaka, Japan), and the number of Cathepsin K-positive cells was counted using BZ X Analyzer software program (KEYENCE, Osaka, Japan).

### Definition of osteoclast positive group and negative group

**Figure 1:**
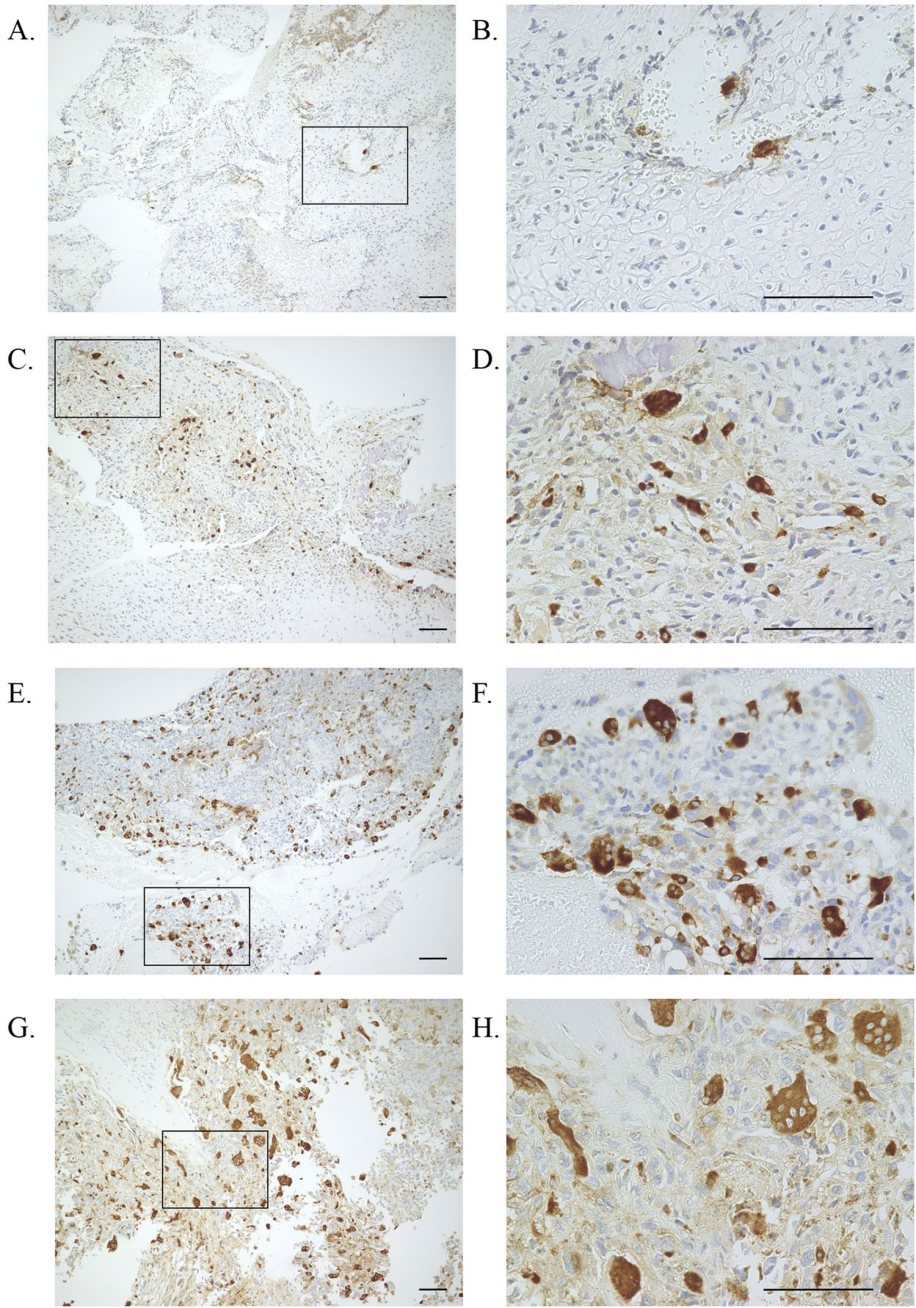
(**A**) Few Cathepsin K-positive cells were observed in a biopsy specimen (Magnification; × 10, Scale bar ; 100 µm). (**B**) Cathepsin K-positive cells with ≥ 3 nuclei were not observed in a marked area of (**A**) . (Magnification; × 40, Scale bar ; 100 µm). (**C**) Cathepsin K-positive cells were observed in a biopsy specimen (Magnification; × 10, Scale bar ; 100 µm). (**D**) The observevd number of Cathepsin K-positive cell with ≥ 3 nuclei was 1 in a marked area of (**C**) . (Magnification; × 40, Scale bar ; 100 µm). (**E**) Numerous Cathepsin K-positive cells were observed in a biopsy specimen (Magnification; × 10, Scale bar ; 100 µm). (**F**) The observed number of Cathepsin K-positive cell with ≥ 3 nuclei were 8 in a marked area of (**E**). (Magnification; × 40, Scale bar ; 100 µm). (**G**) Numerous Cathepsin K-positive cells were observed in a biopsy specimen (Magnification; × 10, Scale bar ; 100 µm). (**H**) The observed number of Cathepsin K-positive cell with ≥ 3 nuclei were 10 in a marked area of (**H**). (Magnification; × 40, Scale bar ; 100 µm).

Among the Cathepsin-K positive cells, a cell with ≥ 3 nuclei was defined as an osteoclast. The total number of osteoclast was visually counted in 10 different fields using microscope at 40 times magnification power in a biopsy specimen. The group of the total number of < 5 osteoclasts in a biopsy specimen was defined as an osteoclast negative group (Fig. 1A–D). The group of the total number of ≥ 5 osteoclasts in a biopsy specimen was defined as an osteoclast positive group (Fig. 1E–H).

### Statistical analyses

The 49 cases were divided into 2 groups, based on a ROC curve analysis. The optimal cut-off value was determined by the maximum Youden index for the ROC curve that predicted a good response to neoadjuvant chemotherapy (≥ 90% necrosis of tumor cells [Grade III/IV]). Univariate and multivariate logistic regression analyses were performed to identify factors that could predict a Grade III/IV response in the resected specimen after neoadjuvant chemotherapy. In the univariate analysis and multivariate analyses, *P*-values of < 0.08 and < 0.05, respectively, were considered to indicate statistical significance.

The patient characteristics were investigated in the two groups divided according to the number of mature osteoclast, which was determined based on a ROC curve analysis. Between the two groups, a non-paired student’s t test for age, tumor size, and course number of each agent of chemotherapy, and a chi-squired test for sex, location, stage, and subtypes were compared. In both tests, *P*-values of < 0.05 was considered to indicate statistical significance.

All statistical analyses were performed using EZR (Saitama Medical Center, Jichi Medical University, Saitama, Japan), which is a graphical user interface for the R software program (The R Foundation for Statistical Computing, Vienna, Austria)^27^.

### Ethics declarations

This study was conducted in accordance with the 1975 Declaration of Helsinki.

### Approval for human experiments

The study was approved by the Ethical Institutional Review Board of the Kanazawa University Hospital (2019–061 (3094)), and written informed consent was obtained from all study participants and/or their parents (in case of children).

### Consent to participate/consent to publish

The consent for partcicipation and publication of the manuscript and the related images from the patients and/or their parents (in case of children) was obtained by the Kanazawa University Hospital.

## Supplementary information

Supplementary Information 1.

Supplementary Information 2.

Supplementary Information 3.

## Data Availability

All data generated or analyzed during the present study are included in this published article.

## Abbreviations

OR: Odds ratio
CI: Confidence interval
IHC: Immunohistochemical
DOX: Doxorubicin
CDDP: Cisplatin
IFO: Ifosfamide
MTX: Methotrexate
ROC: Receiver operating characteristics
AUC: Area under the curve
OS: Overall survival
EFS: Event-free survival
