# Author Correction: The number of osteoclasts in a biopsy specimen can predict the efficacy of neoadjuvant chemotherapy for primary osteosarcoma

**DOI:** 10.1038/s41598-021-88629-2

**Published:** 2021-04-19

**Authors:** Yoshihiro Araki, Norio Yamamoto, Katsuhiro Hayashi, Akihiko Takeuchi, Shinji Miwa, Kentaro Igarashi, Takashi Higuchi, Kensaku Abe, Yuta Taniguchi, Hirotaka Yonezawa, Sei Morinaga, Yohei Asano, Hiroko Ikeda, Takayuki Nojima, Hiroyuki Tsuchiya

**Affiliations:** 1 Department of Orthopaedic Surgery, Graduate School of Medical Sciences, Kanazawa University, 13‑1, Takaramachi, Kanazawa‑city, Ishikawa 920‑8641 Japan; 2 Department of Pathology, Kanazawa University, Kanazawa, Japan

Correction to: *Scientific Reports* 10.1038/s41598-020-80504-w, published online 21 January 2021

The original version of this Article contained errors.

Author names Yoshihiro Araki, Norio Yamamoto, Katsuhiro Hayashi, Akihiko Takeuchi, Shinji Miwa, Kentaro Igarashi, Takashi Higuchi, Kensaku Abe, Yuta Taniguchi, Hirotaka Yonezawa, Sei Morinaga, Yohei Asano, Hiroko Ikeda, Takayuki Nojima, Hiroyuki Tsuchiya were incorrectly given as Y. Araki, N. Yamamoto, K. Hayashi, A. Takeuchi, S. Miwa, K. Igarashi, T. Higuchi, K. Abe, Y. Taniguchi, H. Yonezawa, S. Morinaga, Y. Asano, H. Ikeda, T. Nojima, H. Tsuchiya.

In addition, the Article contains a typographical error in the Results section, in the number of years in the ROC curve analyses for the optimal cut-off values.

“The optimal cut-off values were as follows: age, 47 years old (area under the curve [AUC], 0.59); size, 93 mm (AUC, 0.53); number of osteoclasts in the biopsy specimen, 5 (AUC, 0.75), number of courses of DOX as neoadjuvant chemotherapy, 3 (AUC, 0.63); number of courses of CDDP as neoadjuvant chemotherapy, 3 (AUC, 0.65); number of courses of IFO as neoadjuvant chemotherapy, 4 (AUC, 0.51), and number of courses of MTX as neoadjuvant chemotherapy, 1 (AUC, 0.48) (Table 1).”

now reads:

“The optimal cut-off values were as follows: age, 46 years old (area under the curve [AUC], 0.59); size, 93 mm (AUC, 0.53); number of osteoclasts in the biopsy specimen, 5 (AUC, 0.75), number of courses of DOX as neoadjuvant chemotherapy, 3 (AUC, 0.63); number of courses of CDDP as neoadjuvant chemotherapy, 3 (AUC, 0.65); number of courses of IFO as neoadjuvant chemotherapy, 4 (AUC, 0.51), and number of courses of MTX as neoadjuvant chemotherapy, 1 (AUC, 0.48) (Table 1).”

The number of years has been correctly shown in Table 1.

Furthermore, in Table 2, in the subcategory Stage,

“II/IV”

now reads:

“III/IV”

These errors have now been corrected in the PDF and HTML versions of the Article.

