# Supplementary figures and images for: The number of osteoclasts in a biopsy specimen can predict the efficacy of neoadjuvant chemotherapy for primary osteosarcoma
Source: Sci Rep. 2021 Jan 21;11:1989. doi: 10.1038/s41598-020-80504-w (PMC7820005; doi:10.1038/s41598-020-80504-w)

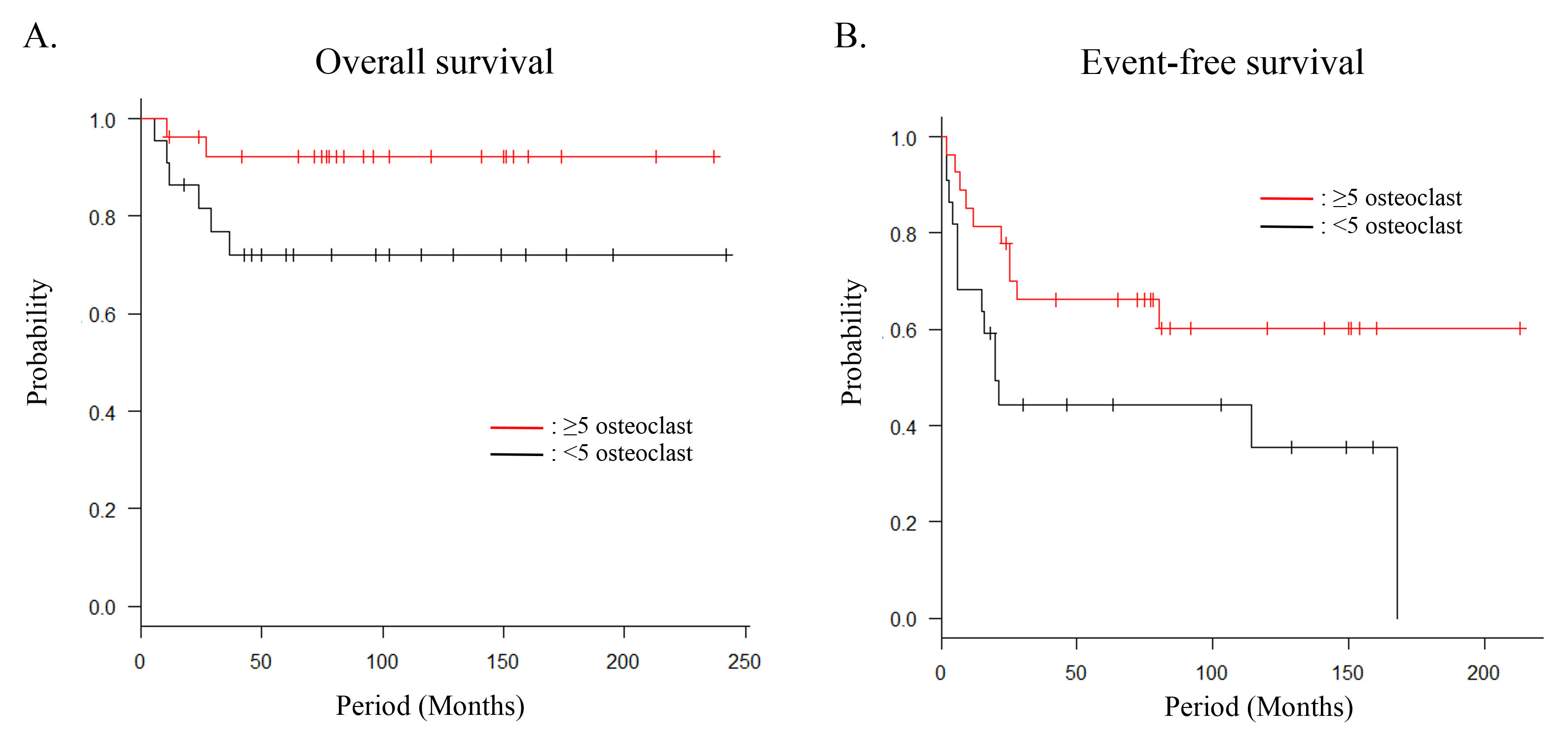

Supplement: Supplementary file 1 — Supplementary Information 1. [file 41598_2020_80504_MOESM1_ESM.tif]
