# Supplementary material for: The number of osteoclasts in a biopsy specimen can predict the efficacy of neoadjuvant chemotherapy for primary osteosarcoma
Source: Sci Rep. 2021 Jan 21;11:1989. doi: 10.1038/s41598-020-80504-w (PMC7820005; doi:10.1038/s41598-020-80504-w)
Supplement: Supplementary file 2 — Supplementary Information 2. [file 41598_2020_80504_MOESM2_ESM.docx]

**Title**

The number of osteoclasts in a biopsy specimen can predict the efficacy of neoadjuvant

chemotherapy for primary osteosarcoma

Araki Y^1^, Yamamoto N^1^, Hayashi K^1^, Takeuchi A^1^, Miwa S^1^, Igarashi K^1^, Higuchi T^1^, Abe K^1^, Taniguchi Y^1^, Yonezawa H^1^, Morinaga S^1^, Asano Y^1^, Ikeda H^2^, Nojima T^2^, Tsuchiya H^1^

**Co-Authors**

Araki Yoshihiro, MD

　y.araki428@gmail.com

<https://ORCID.ORG/0000-0001-5783-109X>

Department of Orthopaedic Surgery, Graduate School of Medical Sciences, Kanazawa University, Kanazawa, Japan

Yamamoto Norio, MD, PhD

norinori@med.kanazawa-u.ac.jp

<https://orcid.org/0000-0002-7250-625X>

Department of Orthopaedic Surgery, Graduate School of Medical Sciences, Kanazawa University, Kanazawa, Japan

Hayashi Katsuhiro, MD, PhD

khayashi830@gmail.com

<https://orcid.org/0000-0001-8665-2154>

Department of Orthopaedic Surgery, Graduate School of Medical Sciences, Kanazawa University, Kanazawa, Japan

Takeuchi Akihiko, MD, PhD

a_take@med.kanazawa-u.ac.jp

<https://orcid.org/0000-0002-4071-5620>

Department of Orthopaedic Surgery, Graduate School of Medical Sciences, Kanazawa University, Kanazawa, Japan

Miwa Shinji, MD, PhD

miwapoti@yahoo.co.jp

<https://orcid.org/0000-0002-5962-8287>

Department of Orthopaedic Surgery, Graduate School of Medical Sciences, Kanazawa University, Kanazawa, Japan

Igarashi Kentaro, MD, PhD

kenken99004@yahoo.co.jp

<https://orcid.org/0000-0003-2278-1736>

Department of Orthopaedic Surgery, Graduate School of Medical Sciences, Kanazawa University, Kanazawa, Japan

Takashi Higuchi, MD, PhD

guchi@384.jp

<https://orcid.org/0000-0002-7489-1657>

Department of Orthopaedic Surgery, Graduate School of Medical Sciences, Kanazawa University, Kanazawa, Japan

Kensaku Abe, MD

abeken.1005@gmail.com

<https://orcid.org/0000-0002-7405-9019>

Department of Orthopaedic Surgery, Graduate School of Medical Sciences, Kanazawa University, Kanazawa, Japan

Taniguchi Yuta, MD

yutataniguchi0925@yahoo.co.jp

<https://orcid.org/0000-0002-4322-6566>

Department of Orthopaedic Surgery, Graduate School of Medical Sciences, Kanazawa University, Kanazawa, Japan

Yonezawa Hirotaka, MD

hirotakayonezawa3@gmail.com

<https://orcid.org/0000-0003-0713-0396>

Department of Orthopaedic Surgery, Graduate School of Medical Sciences, Kanazawa University, Kanazawa, Japan

Morinaga Sei, MD

reddchicke@yahoo.co.jp

<https://orcid.org/0000-0003-2961-9432>

Department of Orthopaedic Surgery, Graduate School of Medical Sciences, Kanazawa University, Kanazawa, Japan

Asano Yohei, MD

you.you.mounin@gmail.com

<https://orcid.org/0000-0002-8777-6076>

Department of Orthopaedic Surgery, Graduate School of Medical Sciences, Kanazawa University, Kanazawa, Japan

Ikeda Hiroko, MD, PhD

[h-ikeda@med.kanazawa-u.ac.jp](mailto:h-ikeda@med.kanazawa-u.ac.jp)

Department of Pathology, Kanazawa University, Kanazawa, Japan

Nojima Takayuki

nojima@kanazawa-med.ac.jp

<https://orcid.org/0000-0003-1236-4162>

Department of Pathology, Kanazawa University, Kanazawa, Japan

Tsuchiya Hiroyuki, MD, PhD

tsuchi@med.kanazawa-u.ac.jp

<https://orcid.org/0000-0003-0730-7921>

Department of Orthopaedic Surgery, Graduate School of Medical Sciences, Kanazawa University, Kanazawa, Japan

1. Department of Orthopaedic Surgery, Graduate School of Medical Sciences, Kanazawa University, Kanazawa, Japan
2. Department of Pathology, Kanazawa University, Kanazawa, Japan

**Corresponding author**

Norio Yamamoto, MD, PhD

Department of Orthopaedic Surgery, Graduate school of medical sciences, Kanazawa University, Kanazawa, Japan

13-1, Takaramachi, Kanazawa-city, Ishikawa, 920-8641, Japan

TEL: 81-76-265-2000

E-mail: norinori@med.kanazawa-u.ac.jp

<https://orcid.org/0000-0002-7250-625X>

**Supplementary Figure Legend**

Supplementary Figure S1.

A) Overall survival of osteosarcoma patients who underwent neoadjuvant chemotherapy according to the presence (≥5) or absence (<5) of osteoclasts.

B) Event-free survival of osteosarcoma patients who underwent neoadjuvant chemotherapy according to the presence (≥5) or absence (<5) of osteoclasts.
