# Supplementary material for: The number of osteoclasts in a biopsy specimen can predict the efficacy of neoadjuvant chemotherapy for primary osteosarcoma
Source: Sci Rep. 2021 Jan 21;11:1989. doi: 10.1038/s41598-020-80504-w (PMC7820005; doi:10.1038/s41598-020-80504-w)
Supplement: Supplementary file 3 — Supplementary Information 3. [file 41598_2020_80504_MOESM3_ESM.docx]

**Title**

The number of osteoclasts in a biopsy specimen can predict the efficacy of neoadjuvant

chemotherapy for primary osteosarcoma

Araki Y^1^, Yamamoto N^1^, Hayashi K^1^, Takeuchi A^1^, Miwa S^1^, Igarashi K^1^, Higuchi T^1^, Abe K^1^, Taniguchi Y^1^, Yonezawa H^1^, Morinaga S^1^, Asano Y^1^, Ikeda H^2^, Nojima T^2^, Tsuchiya H^1^

**Co-Authors**

Araki Yoshihiro, MD

　y.araki428@gmail.com

<https://ORCID.ORG/0000-0001-5783-109X>

Department of Orthopaedic Surgery, Graduate School of Medical Sciences, Kanazawa University, Kanazawa, Japan

Yamamoto Norio, MD, PhD

norinori@med.kanazawa-u.ac.jp

<https://orcid.org/0000-0002-7250-625X>

Department of Orthopaedic Surgery, Graduate School of Medical Sciences, Kanazawa University, Kanazawa, Japan

Hayashi Katsuhiro, MD, PhD

khayashi830@gmail.com

<https://orcid.org/0000-0001-8665-2154>

Department of Orthopaedic Surgery, Graduate School of Medical Sciences, Kanazawa University, Kanazawa, Japan

Takeuchi Akihiko, MD, PhD

a_take@med.kanazawa-u.ac.jp

<https://orcid.org/0000-0002-4071-5620>

Department of Orthopaedic Surgery, Graduate School of Medical Sciences, Kanazawa University, Kanazawa, Japan

Miwa Shinji, MD, PhD

miwapoti@yahoo.co.jp

<https://orcid.org/0000-0002-5962-8287>

Department of Orthopaedic Surgery, Graduate School of Medical Sciences, Kanazawa University, Kanazawa, Japan

Igarashi Kentaro, MD, PhD

kenken99004@yahoo.co.jp

<https://orcid.org/0000-0003-2278-1736>

Department of Orthopaedic Surgery, Graduate School of Medical Sciences, Kanazawa University, Kanazawa, Japan

Takashi Higuchi, MD, PhD

guchi@384.jp

<https://orcid.org/0000-0002-7489-1657>

Department of Orthopaedic Surgery, Graduate School of Medical Sciences, Kanazawa University, Kanazawa, Japan

Kensaku Abe, MD

abeken.1005@gmail.com

<https://orcid.org/0000-0002-7405-9019>

Department of Orthopaedic Surgery, Graduate School of Medical Sciences, Kanazawa University, Kanazawa, Japan

Taniguchi Yuta, MD

yutataniguchi0925@yahoo.co.jp

<https://orcid.org/0000-0002-4322-6566>

Department of Orthopaedic Surgery, Graduate School of Medical Sciences, Kanazawa University, Kanazawa, Japan

Yonezawa Hirotaka, MD

hirotakayonezawa3@gmail.com

<https://orcid.org/0000-0003-0713-0396>

Department of Orthopaedic Surgery, Graduate School of Medical Sciences, Kanazawa University, Kanazawa, Japan

Morinaga Sei, MD

reddchicke@yahoo.co.jp

<https://orcid.org/0000-0003-2961-9432>

Department of Orthopaedic Surgery, Graduate School of Medical Sciences, Kanazawa University, Kanazawa, Japan

Asano Yohei, MD

you.you.mounin@gmail.com

<https://orcid.org/0000-0002-8777-6076>

Department of Orthopaedic Surgery, Graduate School of Medical Sciences, Kanazawa University, Kanazawa, Japan

Ikeda Hiroko, MD, PhD

[h-ikeda@med.kanazawa-u.ac.jp](mailto:h-ikeda@med.kanazawa-u.ac.jp)

Department of Pathology, Kanazawa University, Kanazawa, Japan

Nojima Takayuki

nojima@kanazawa-med.ac.jp

<https://orcid.org/0000-0003-1236-4162>

Department of Pathology, Kanazawa University, Kanazawa, Japan

Tsuchiya Hiroyuki, MD, PhD

tsuchi@med.kanazawa-u.ac.jp

<https://orcid.org/0000-0003-0730-7921>

Department of Orthopaedic Surgery, Graduate School of Medical Sciences, Kanazawa University, Kanazawa, Japan

1. Department of Orthopaedic Surgery, Graduate School of Medical Sciences, Kanazawa University, Kanazawa, Japan
2. Department of Pathology, Kanazawa University, Kanazawa, Japan

**Corresponding author**

Norio Yamamoto, MD, PhD

Department of Orthopaedic Surgery, Graduate school of medical sciences, Kanazawa University, Kanazawa, Japan

13-1, Takaramachi, Kanazawa-city, Ishikawa, 920-8641, Japan

TEL: 81-76-265-2000

E-mail: norinori@med.kanazawa-u.ac.jp

<https://orcid.org/0000-0002-7250-625X>

**Supplementary Methods**

**Statistical analyses for survival**

The overall survival and event-free survival were determined by a Kaplan-Meir curve analysis, and were compared using a log-rank test between the two groups divided according to the presence or absence of osteoclasts in a biopsy specimen. Overall survival was considered as the time until death from the diagnosis of osteosarcoma. Event-free survival was considered as the time until the development of relapse or metastasis at the other sites from the time of enrollment. The statistical analyses were performed using EZR (Saitama Medical Center, Jichi Medical University, Saitama, Japan), which is a graphical user interface for the R software program (The R Foundation for Statistical Computing, Vienna, Austria) ^27^.
